# Haplotype of gene Nedd4 binding protein 2 associated with sporadic nasopharyngeal carcinoma in the Southern Chinese population

**DOI:** 10.1186/1479-5876-5-36

**Published:** 2007-07-13

**Authors:** Mei-Zhen Zheng, Hai-De Qin, Xing-Juan Yu, Ru-Hua Zhang, Li-Zhen Chen, Qi-Sheng Feng, Yi-Xin Zeng

**Affiliations:** 1 State Key Laboratory of Oncology in Southern China and Department of Experimental Research, Sun Yat-sen University Cancer Center, 651 Dongfeng Road East, Guangzhou, China

## Abstract

**Background:**

Bcl-3 as an oncoprotein is overexpressed in nasopharyngeal carcinoma (NPC). Nedd4 binding protein 2 (N4BP2), which is located in the NPC susceptibility locus, is a Bcl-3 binding protein. This study is aimed to explore the association between N4BP2 genetic polymorphism and the risk of NPC.

**Methods:**

We performed a hospital-based case-control study, including 531 sporadic NPC and 480 cancer-free control subjects from southern China. PCR-sequencing was carried out on Exons, promoter region and nearby introns of the N4BP2 gene. The expression pattern of N4BP2 and Bcl-3 was also analyzed.

**Results:**

We observed a statistically significant difference in haplotype blocks ATTA and GTTG between cases and controls. In addition, three novel SNPs were identified, two of which were in exons (loc123-e3l-snp2, position 39868005, A/G, Met171Val; RS17511668-SNP2, position 39926432, G/A, Glu118Lys), and one was in the intron6 (RS794001-SNP1, position 39944127, T/G). Moreover, N4BP2 was at higher levels in a majority of tumor tissues examined, relative to paired normal tissues.

**Conclusion:**

These data suggest that haplotype blocks ATTA and GTTG of N4BP2 is correlation with the risk of sporadic nasopharyngeal carcinoma in the Southern Chinese population and N4BP2 has a potential role in the development of NPC.

## Background

Bcl-3 was originally identified as expressed in chronic B cell lymphocytic leukemia [1]. Many cell growth and survival promoters can induce Bcl-3 expression, and Bcl-3 overexpression has been detected in other cancers such as nasopharyngeal carcinoma (NPC) [2,3]. Nedd4 binding protein 2 (N4BP2, GenBank:AY267013) is a Bcl-3 binding protein, N4BP2 protein contains a polynucleotide kinase domain (PNK) at the N-terminus and a Small MutS Related (Smr) domain with nicking endonuclease activity near C-terminus [3]. MutS is central to the DNA mismatch repair (MMR) systems that are responsible for maintaining genome stability and protecting against mutation, Mutations in these genes are linked to the development of certain types of cancer [4,5]. Since N4BP2 contains a MutS-related domain, N4BP2 may play a role in MMR.

NPC is an epithelial tumor with an exceptionally high incidence in southern China, particularly in the Guangdong province [6,7]. Etiological and epidemiological studies have suggested that susceptibility genes may determine the predisposition to developing NPC [8,9]. Previously, we reported the use of 382 polymorphic microsatellite markers to identify a candidate susceptibility locus that mapped to chromosome 4p15.1-q12 (D4S2950-D4S2916) in a subset of NPC families [10]. Further analysis identified SNPs within or near this region, strongly suggesting the presence of an NPC susceptibility locus adjacent to the LOC344967 [11], very close to the *N4BP2 *gene.

We thus hypothesized that SNPs or other variation in the *N4BP2 *gene lead to a predisposition to developing NPC. We further hypothesized that the *N4BP2 *gene plays a role in tumorigenesis. To address these hypotheses, we examined *N4BP2 *haplotypes among NPC patients from southern China. We also examined mRNA levels of *Bcl-3 *and *N4BP2 *in NPC cell lines and tissues.

## Methods

### Subjects

A total of 531 sporadic NPC patients and 480 unrelated age, sex-, and geographically-matched healthy individuals from southern China were used for our case-control study. NPC patients were recruited from Sun Yat-sen University Cancer Center and the presence of differentiated non-keratinizing NPC or undifferentiated NPC was confirmed by histological analysis. Control subjects were recruited from the People's Hospital of Guangdong Province. The characteristics of cases and controls are shown in Table 1. Although the incidence of NPC is generally higher among males than females, no significant difference in sex distribution existed between case and control groups in this study. The average age in the control group was 37 ± 10, and in the case group was 40 ± 10; thus the age distribution here is a good representation of the broader NPC patient population given that NPC incidence peaks at the relatively young age of 45.

**Table 1:** Profile of the study subjects

Characteristic	Control (n = 480)	Case (n = 531)	Chi square	*p*^a^
Gender (Male/Female)	354/126	390/141	0.01	0.91
Age	37 ± 10	40 ± 10	0.86	0.44

^a^*p *value obtained from Chi-square Test

### DNA preparation

Genomic DNA was extracted from 5–10 ml peripheral blood using the QIAamp DNA Blood Midi kit (Qiagen, German).

### Primer design

Between 250 and 400 bp of sequence surrounding SNPs sites were submitted to the primer design program [12]. The primers used for SNPs are shown in Table 2.

**Table 2:** Primers used for SNPs

Number	RefSNP(RS) (NCBI)	Forward primers(5'→3')	Reverse primer(5'→3')	Product size (bp)
SNP1	RS17439810	agctgacagtgttcggctct	ggtcaacatgatgtccgaaa	257
SNP2	loc123-e3l-snp2^a^			
SNP3	RS17511578	cttctcgtcctgggtctcc	TGTCAGCTACAGCCAGTCCA	397
SNP4	RS17585937	acatgggccacaggaagtg	aggcctgccagacacctg	266
SNP6	RS17511668-SNP2^b^	TCTGTTCAATGCAAAATCCAA	catcttGTGAAGGCAAAAATGA	389
SNP7	RS794001-SNP1^c^	GCTTCATATATGCCAAGCATCA	TTTTCTTCTTTTGTTTAAATCTGCATT	398
SNP9	RS2252352	agaTGCAGCTCAATCACCTA	CAGAAGAAATTGCCTTACAGGA	300
SNP10	RS2271395	tccagtctagtcaaaatggtgaga	aacacacagtgcaatttcttaactg	243
SNP11	RS1442855	TCATTTGCTTCAGCTTGTCA	GGCCTTAAACCTTACTCCATCC	300
SNP12	RS2347044	TCTTGTGGTGAAATGTAGAATGC	CCTGAGATTAACTACCCATATCAGC	368

^a ^Primers for loc123-e3l-snp2 are same as RS17439810.

^b ^primers for RS17511668-SNP2 are same as RS17511668

^c ^RS794001-SNP1 are same as RS794001.

### PCR Amplification

Long-distance PCR was performed in a total volume of 15 μl containing 200 ng of genomic DNA, 1.5 μl 10× Buffer, 50 μM dNTPs, 0.3 μM each primer, and 1U Taq DNA polymerase. Samples were amplified with each pair of primers described above as follows: 94°C for 3 min, 10 cycles of 94°C for 30 s, 63°C for 1 min, and 72°C for 1 min; 25 cycles of 94°C for 30 s, 58°C for 1 min, 72°C for 1 min, and a final extension at 72°C for 7 min. First-round PCR products were diluted 5-fold for the second-round of PCR. Round 2 PCR conditions were 94°C for 3 min, 10 cycles at 94°C for 30 s, 65°C for 1 min, and 72°C for 1 min; 30 cycles at 94°C for 30 s, 62°C for 1 min, and 72°C for 1 min, and 72°C for 7 min. PCR products were visualized on a 1.2% agarose gel, stained with ethidium bromide, and visualized by a transilluminator.

### Genotyping

PCR products were sequence using ABI377 or ABI3730 sequencers (PE Applied biosystem). Base calling, contig assembly contigs, and mutation detection was performed using Polyphred package (Polyphred, Phred/Phrap/Consed) [13]. All traces were visually inspected by at least two observers.

### Statistical methods

Unrelated control samples were selected for analysis using the Hardy-Weinberg Equilibrium (HWE) test using an exact test. Standard EM algorithm was used to infer haplotype and estimate population frequency. Single marker and haplotype association test and significance estimation were performed using a permutation test.

### Cell Culture and Treatment

NP69 (an SV40 large T antigen-immortalized nasopharyngeal epithelial cell line) and NP69-LMP1 (NP69 cells transfected with the *LMP1 *gene) were cultured in Keratinocyte-SFM medium (GibcoBRL) with Bovine Pituitary Extract and rEGF. C666-1 (a poorly differentiated nasopharyngeal epithelial cell line carrying the Epstein-Barr virus) was grown in RPMI 1640 supplemented with 10% fetal bovine serum (FBS, Hyclone, Utah, USA). CNE-1 (a highly-differentiated nasopharyngeal epithelial cell line), CNE-2 (a poorly differentiated nasopharyngeal epithelial cell line) and Sune(a poorly differentiated nasopharyngeal epithelial cell line carried Epstein-Barr virus) were maintained in RPMI 1640 with 10% FBS.

### Tissue collection and RT-PCR

A total of 21 tissues were collected from Sun Yat-sen University Cancer Center. Six paired matched tissues from different organs included esophagus, stomach, liver, lung, cervix and breast; nine nasopharyngeal tissues contained 2 chronic nasopharynx inflammation (Inf.), 1 Differentiated Carcinoma (DNK), 4 Undifferentiated Carcinoma (NDNK), 1 low differentiated squamous carcinoma (LDS) and 1 non-Hodgkin's lymphoma (NHL). RNA was extracted using TRIZOL Reagent (Invitrogen, Carlsbad, CA), and reverse transcription was performed using the TaKaRa RNA PCR kit (AMV) Ver.3.0 (TaKaRa BIO, Shiga, Japan). PCR to detect *N4BP2 *was performed using the following primers (*N4BP2-L*: 5'-AAAGGGAGACCCTTATGTTTGA-3'; *N4BP2-R*: 5'-AAATCAAACCTCACTTGCATTT-3') and *Bcl*-3 with primers (*Bcl-3-L*:5'-tcctctggtgaacctgccta-3'; *Bcl-3-R*:5'-gaagaccattggagctgagg-3') and *β-actin *as control with primers (5'-acactgtgcccatctacgagggg-3' and 5'-atgatggagttgaaggtagtttcgtggat-3').

## Results

### SNP analysis

We identified a total of twelve SNP associated with the *N4BP2 *locus, four of which were upstream of the *N4BP2 *gene and eight which were within *N4BP2 *gene (Table 3). Of the SNPs, five SNPs resulted in missense mutations. Three novel SNPs were identified: loc123-e3l-snp2 (position 39868005, allele A/G, resulting in amino acid change Met171 to Val), RS17511668-SNP2 (position 39926432, allele G/A, amino acid change Glu118 to Lys), and RS794001-SNP1 (position 39944127, allele T/G, intronic). However, allele frequency analysis revealed no significant difference between case and control groups (Table 4).

**Table 3:** Candidate SNPs

Number	RefSNP	Contig Position	Variant Alleles	Amino acid change	MAF	HWpval	Genotyped (%)
SNP1	RS17439810	39867905	C, G	Gln104Glu	0.058	0.195	74.0
**SNP2**	**loc123-e3l-snp2**	**39868005**	**A, G**	**Met171Val**	**0.271**	**0.999**	**81.4**
SNP3	RS17511578	39868252	A, C	Asn189His	0.063	0.978	74.8
SNP4	RS17585937	39868402	G, A	5'UTR	0.118	1.000	76.2
**SNP6**	**RS17511668-SNP2**	**39926432**	**G, A**	**Glu118Lys**	**0.067**	**1.000**	**89.9**
**SNP7**	**RS794001-SNP1**	**39944127**	**T, G**	**intron**	**0.054**	**0.562**	**97.4**
SNP9	RS2252352	39950655	T, T	intron	0.368	0.327	96.1
SNP10	RS2271395	39961241	G, A	Ala1587Thr	0.368	1.000	94.8
SNP11	RS1442855	43164843	G, A	intron	0.164	0.946	82.5
SNP12	RS2347044	43273849	A, G	intron	0.225	0.377	96.9

RefSNP(RS), SNP accession ID; HWpval, *P *value of Hardy-Weinberg equilibrium tests for control subjects; MAF, minor allele frequency; % Genotyped, Percent of samples successfully genotyped. New SNPs are shown in bold.

**Table 4:** SNP allele frequency comparison between cases and controls

Number	Name	Major Alleles	Case, Control Ratios^a^	Chi square	P value
SNP1	loc123-e3l-snp1	C, C	772:42 452:34	1.864	0.1722
SNP2	loc123-e3l-snp2	A, A	570:224 472:164	1.051	0.3053
SNP3	loc123-e3l-snp3	G, G	659:51 572:32	1.960	0.1615
SNP4	LOC344967	G, G	634:74 546:84	2.658	0.1031
SNP6	RS17511668-SNP2	G, G	802:66 670:40	2.419	0.1199
SNP7	RS794001-SNP1	T, T	890:48 728:44	0.282	0.5954
SNP9	RS2252352	T, T	574:320 493:301	0.809	0.3684
SNP 10	RS2271395	A, A	599:331 452:282	1.410	0.2350
SNP 11	RS1442855	G, G	646:128 565:109	0.035	0.8513
SNP 12	RS2347044	A, A	712:212 607:171	0.225	0.6351

^a ^Case, Control Ratios = the frequency of Major Alleles: the frequency of Minor Alleles

### Haplotype analysis

Haplotype frequencies and distributions were estimated using a standard EM algorithm. Interestingly, Four SNPs (SNP6-7-9-10) combined haplotype Block 2 ATTA and GTTG exhibited notable difference between case and control groups (Table 5). Permutation tests for allelic association confirmed that block ATTA and GTTG are closely linked (Table 6) and confirmed the difference of Block 2 ATTA and GTTG in cases and controls.

**Table 5:** Haplotype analysis

Block	Haplotype	Frequencies^a^	Case, Control Ratios^b^	Chi Square	P Value
1(SNP1-4)					
	CAG	0.55	519.0:411.0, 420.2:357.8	0.557	0.4556
	CGG	0.272	261.2:668.8, 204.2:573.8	0.724	0.3947
	CAA	0.119	100.6:829.4, 103.0:675.0	2.368	0.1238
	GAG	0.058	49.2:880.8, 50.6:727.4	1.149	0.2837
2(SNP6-7-9-10)					
	GTTA	0.542	521.4:426.6, 429.0:377.0	0.549	0.4589
	GTCG	0.355	328.2:619.8, 294.3:511.7	0.684	0.4082
	AGTA	0.047	40.4:907.6, 41.6:764.4	0.806	0.3692
	**ATTA**	**0.017**	**27.4:920.6, 2.7:803.3**	**16.903**	**3.93E-05**
	**GTTG**	**0.016**	**4.6:943.4, 23.3:782.7**	**16.038**	**6.21E-05**
	GTCA	0.015	13.1:934.9, 12.9:793.1	0.14	0.7079

Bold type indicates significant difference between case and control.

^a ^frequency of haplotype in total subjects.

^b^frequency of haplotype: frequency of non-haplotype.

**Table 6:** Permutation test

Name	Chi Square	Permutation p-value
**Block 2: ATTA**	**16.903**	**0.0007**
**Block 2: GTTG**	**16.038**	**0.0013**
Block 1: CAA	2.368	0.8602
Block 1: GAG	1.149	0.9916
Block 2: AGTA	0.806	0.9992
Block 1: CGG	0.724	0.9997
Block 2: GTCG	0.684	0.9998
Block 1: CAG	0.557	1
Block 2: GTTA	0.549	1
Block 2: GTCA	0.14	1
		
LOC344967	2.658	0.8071
RS17511668-SNP2	2.419	0.8498
loc123-e3l-snp3	1.96	0.9218
loc123-e3l-snp1	1.864	0.9366
RS2271395	1.41	0.9807
loc123-e3l-snp2	1.051	0.9952
RS2252352	0.809	0.9991
RS794001-SNP1	0.282	1
RS1442855	0.035	1
RS2347044	0.225	1
10000 permutations performed.		

Haplotype in bold is closely linked block

### N4BP2 and Bcl-3 expressed in cells and tissues

**Figure 1:**
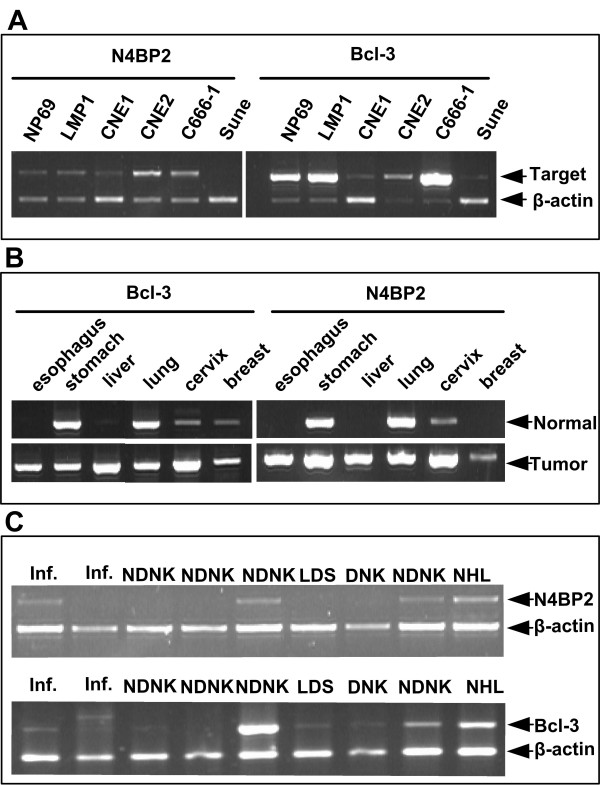
**Expression of *N4BP2 *and *Bcl-3 *mRNA in cells and tissues**. (A) Expression of *N4BP2 *and *Bcl-3 *mRNA in nasopharyngeal epithelial cell lines. RT-PCR was performed with gene-specific primers and *β-actin *was used as a control. (B) Expression of *N4BP2 *and *Bcl-3 *mRNA in matched tissues. RT-PCR was performed with gene-specific primers and *β-actin *as a control. up-low: normal tissue; down-low: tumor tissue. (C) Expression of *N4BP2 *and *Bcl-3 *mRNA nasopharyngeal tissues. Inf.: chronic nasopharynx inflammation; NDNK: Undifferentiated Carcinoma; DNK: Differentiated Carcinoma; LDS: Low differentiated squamous carcinoma; NHL: non-Hodgkin's lymphoma.

*Bcl-3 *and *N4BP2 *were detected in all cell lines examined (Figure 1A). Interestingly, gene expression levels appeared to vary among the cell lines, with the lowest levels being detected in the Sune line. *N4BP2 *and *Bcl-3 *mRNA levels appeared to be higher in tumor than in matched normal tissue (Figure 1B). *N4BP2 *and *Bcl-3 *were also detected in nasopharyngeal tissues (Figure 1C). These observations suggest that *N4BP2 *expression levels correlate with the progression of cancer including NPC.

## Discussion

We previously showed, by linkage analysis that an NPC susceptibility locus maps to chromosome 4 near the *LOC344967*. Here, we extend this analysis in an effort to identify a bona fide NPC susceptibility gene. *N4BP2 *is a candidate gene in this region, and we thus sought to examine the correlation between genetic polymorphisms in *N4BP2*, a mismatch repair gene, and the incidence of NPC. SNPs have been shown to be extremely useful for studying the association between genomic regions and disease [14]. Several studies have demonstrated that polymorphic variation in mismatch repair genes contributes to susceptibility to certain cancers [15,16]. In addition, several pieces of evidence suggest that nasopharyngeal carcinogenesis is associated with individual susceptibility caused by SNPs. Our results show that, although there is no significant difference in SNPs between cases and controls, there are two haplotypes, ATTA and GTTG, the distribution of which differed between case and control groups. Just as the report that almost not any difference in the allele frequencies of five SNPs within the *TNFSF4 *gene individuals suffered from coronary artery disease versus the controls while there were significantly more frequent of the possible haplotypes from this five TNFSF4 SNPs in individuals with coronary artery disease than controls [17]. Insight into this paradox has been provided in a recent review by Schaid [18]. Haplotypes, the grouping of closely linked alleles on a chromosome, make an important contribution to the study of the genetic basis of disease. Schaid [18] explained that for case-control studies, methodological approach based on haplotype has more advantage than single-locus analysis as the SNPs are in LD with a causative diallelic locus; in particular, haplotype-based methods have greater power when the marker alleles are in strong LD with causative alleles. Haplotype methods are more useful for variants which are more recently evolved, rarer and more causative than for variants which are older and more common. This may help explain why the haplotypes ATTA and GTTG exhibit differences in frequency between case and control groups while individual SNPs do not.

N4BP2 is a Bcl-3 binding protein, and Bcl-3 is an oncoprotein that is overexpressed in certain cancers, including NPC. Our analysis of *N4BP2 *and *Bcl-3 *expression levels suggest that expression of these genes is correlated, suggesting they may be co-regulated. We also found that *N4BP2 *and *Bcl-3 *are expressed in all NPC cell lines examined and were higher for certain cancers. These observations are consistent with previous reports.

## Conclusion

Above all, we found that two *N4BP2 *haplotypes, ATTA and GTTG, are correlated with NPC. This will be useful for predict the development of NPC. In addition, *N4BP2 *and *Bcl-3 *mRNA levels were elevated in tumors, including NPC tumors, which suggest new therapeutic targets for fighting NPC.

## Competing interests

The author(s) declare that they have no competing interests.

## Authors' contributions

YXZ and HDQ were responsible for the design of study. MZZ carried out the experiments and drafted the manuscript. HDQ participated in data analysis. XJY and RHZ performed sequencing. LZC and QSF helped in cell culture and tissue collection.

## References

[B1] McKeithan T, Ohno H, Rowley J, Diaz M (1989). Cloning of the breakpoint junction of the translocation 14;19 in chronic lymphocytic leukemia. Haematol Blood Transfus.

[B2] Thornburg NJ, Pathmanathan R, Raab-Traub N (2003). Activation of nuclear factor-kappaB p50 homodimer/Bcl-3 complexes in nasopharyngeal carcinoma. Cancer Res.

[B3] Watanabe N, Wachi S, Fujita T (2003). Identification and characterization of BCL-3-binding protein: implications for transcription and DNA repair or recombination. J Biol Chem.

[B4] Lamers MH, Perrakis A, Enzlin JH, Winterwerp HH, de Wind N, Sixma TK (2000). The crystal structure of DNA mismatch repair protein MutS binding to a G × T mismatch. Nature.

[B5] Obmolova G, Ban C, Hsieh P, Yang W (2000). Crystal structures of mismatch repair protein MutS and its complex with a substrate DNA. Nature.

[B6] Chen DL, Huang TB (1997). A case-control study of risk factors of nasopharyngeal carcinoma. Cancer Lett.

[B7] Hsu JL, Glaser SL (2000). Epstein-barr virus-associated malignancies: epidemiologic patterns and etiologic implications. Crit Rev Oncol Hematol.

[B8] Hildesheim A, Dosemeci M, Chan CC, Chen CJ, Cheng YJ, Hsu MM, Chen IH, Mittl BF, Sun B, Levine PH, Chen JY, Brinton LA, Yang CS (2001). Occupational exposure to wood, formaldehyde, and solvents and risk of nasopharyngeal carcinoma. Cancer Epidemiol Biomarkers Prev.

[B9] Cheng YJ, Hildesheim A, Hsu MM, Chen IH, Brinton LA, Levine PH, Chen CJ, Yang CS (1999). Cigarette smoking, alcohol consumption and risk of nasopharyngeal carcinoma in Taiwan. Cancer Causes Control.

[B10] Feng BJ, Huang W, Shugart YY, Lee MK, Zhang F, Xia JC, Wang HY, Huang TB, Jian SW, Huang P, Feng QS, Huang LX, Yu XJ, Li D, Chen LZ, Jia WH, Fang Y, Huang HM, Zhu JL, Liu XM, Zhao Y, Liu WQ, Deng MQ, Hu WH, Wu SX, Mo HY, Hong MF, King MC, Chen Z, Zeng YX (2002). Genome-wide scan for familial nasopharyngeal carcinoma reveals evidence of linkage to chromosome 4. Nat Genet.

[B11] Jiang RC, Qin HD, Zeng MS, Huang W, Feng BJ, Zhang F, Chen HK, Jia WH, Chen LZ, Feng QS, Zhang RH, Yu XJ, Zheng MZ, Zeng YX (2006). A functional variant in the transcriptional regulatory region of gene LOC344967 cosegregates with disease phenotype in familial nasopharyngeal carcinoma. Cancer Res.

[B12] Primer3. http://www-genome.wi.mit.edu/cgi-bin/primer/primer3_www.cgi.

[B13] Department of genome sciences university of Washington. http://droog.gs.washington.edu/.

[B14] Kwok PY, Gu Z (1999). Single nucleotide polymorphism libraries: why and how are we building them?. Mol Med Today.

[B15] Kawakami T, Shiina H, Igawa M, Deguchi M, Nakajima K, Ogishima T, Tokizane T, Urakami S, Enokida H, Miura K, Ishii N, Kane CJ, Carroll PR, Dahiya R (2004). Inactivation of the hMSH3 mismatch repair gene in bladder cancer. Biochem Biophys Res Commun.

[B16] Apessos A, Mihalatos M, Danielidis I, Kallimanis G, Agnantis NJ, Triantafillidis JK, Fountzilas G, Kosmidis PA, Razis E, Georgoulias VA, Nasioulas G (2005). hMSH2 is the most commonly mutated MMR gene in a cohort of Greek HNPCC patients. Br J Cancer.

[B17] Wang X, Ria M, Kelmenson PM, Eriksson P, Higgins DC, Samnegård A, Petros C, Rollins J, Bennet AM, Wiman B, de Faire U, Wennberg C, Olsson PG, Ishii N, Sugamura K, Hamsten A, Forsman-Semb K, Lagercrantz J, Paigen B (2005). Positional identification of TNFSF4, encoding OX40 ligand, as a gene that influences atherosclerosis susceptibility. Nat Genet.

[B18] Schaid DJ (2004). Evaluating associations of haplotypes with traits. Genet Epidemiol.

